# Brachytherapy in a Single Dose of 10Gy as an “in situ” Vaccination

**DOI:** 10.3390/ijms21134585

**Published:** 2020-06-28

**Authors:** Magdalena Jarosz-Biej, Ryszard Smolarczyk, Tomasz Cichoń, Alina Drzyzga, Justyna Czapla, Zbigniew Urbaś, Ewelina Pilny, Sybilla Matuszczak, Piotr Wojcieszek

**Affiliations:** 1 Center for Translational Research and Molecular Biology of Cancer, Maria Sklodowska-Curie National Research Institute of Oncology, Gliwice Branch, Wybrzeże Armii Krajowej Street 15, 44-101 Gliwice, Poland; Ryszard.Smolarczyk@io.gliwice.pl (R.S.); Tomasz.Cichon@io.gliwice.pl (T.C.); Alina.Hadyk@io.gliwice.pl (A.D.); Justyna.Czapla@io.gliwice.pl (J.C.); Ewelina.Pilny@io.gliwice.pl (E.P.); Sybilla.Matuszczak@io.gliwice.pl (S.M.); 2 Brachytherapy Department, Maria Sklodowska-Curie National Research Institute of Oncology, Gliwice Branch, Wybrzeże Armii Krajowej Street 15, 44-101 Gliwice; Poland; zbigniew87@gmail.com (Z.U.); Piotr.Wojcieszek@io.gliwice.pl (P.W.)

**Keywords:** brachytherapy, dose, *“in situ”* vaccination, tumor vasculature, tumor microenvironment

## Abstract

Radiotherapy (RT) is one of the major methods of cancer treatment. RT destroys cancer cells, but also affects the tumor microenvironment (TME). The delicate balance between immunomodulation processes in TME is dependent, among other things, on a specific radiation dose. Despite many studies, the optimal dose has not been clearly determined. Here, we demonstrate that brachytherapy (contact radiotherapy) inhibits melanoma tumor growth in a dose-dependent manner. Doses of 10Gy and 15Gy cause the most effective tumor growth inhibition compared to the control group. Brachytherapy, at a single dose of ≥ 5Gy, resulted in reduced tumor blood vessel density. Only a dose of 10Gy had the greatest impact on changes in the levels of tumor-infiltrating immune cells. It most effectively reduced the accumulation of protumorogenic M2 tumor-associated macrophages and increased the infiltration of cytotoxic CD8^+^ T lymphocytes. To summarize, more knowledge about the effects of irradiation doses in anticancer therapy is needed. It may help in the optimization of RT treatment. Our results indicate that a single dose of 10Gy leads to the development of a robust immune response. It seems that it is able to convert a tumor microenvironment into an “in situ” vaccine and lead to a significant inhibition of tumor growth.

## 1. Introduction

Radiotherapy (RT) is one of the main treatment methods for many types of cancer. More than half of all oncological patients undergo radiation treatment during their therapy [1]. For a long time, RT has been used to treat melanoma patients [2]. Melanoma is one of the most common skin cancers. In 2016, in the United States the estimated number of newly diagnosed patients suffering from melanoma reached 76,380 [3]. The US Surveillance, Epidemiology, and End Results data have shown that the incidence of melanoma has increased four times in the last four decades and risen 1.5% annually in the last 10 years [4]. Brachytherapy (contact radiotherapy) is used in the treatment of skin cancer [5]. It involves the use of a radioactive source directly adjacent to the malignant tissue. The advantage of this technique is the administration of a high dose radiation locally to treated lesions. Such a treatment reduces toxicity when compared to external beam radiotherapy. Brachytherapy also enables a reduction in the radiation dose in exposed adjacent normal structures [6].

Irradiation induces a number of different types of cell death, including apoptosis, mitotic catastrophe, autophagy, necroptosis, and necrosis [7]. The type of cancer cell death induced depends on its genetic background, the radiation dose, and the composition of the tumor microenvironment (TME) [8]. TME is a key player in mediating radiotherapy effectiveness. Immunomodulatory processes occurring in TME may be involved in the activation of the immune system, which may contribute to the effect of an “in situ” vaccine. On the other hand, TME-stimulated immunosuppression may lead to radioresistance. There is no conclusive data indicating which dose of radiation affects the activation of the anti-tumor immune response and which one contributes to immunosuppression. The literature data show contradictory information—both low-dose radiation (LDR) and high-dose radiation (HDR) can activate and also inhibit the antitumor immune response [9].

Therefore, as a part of our research we focused on the effect of various doses of brachytherapy—2Gy, 5Gy, 10Gy, and 15Gy—on the inhibition of B16-F10 murine melanoma tumor growth. We checked how selected doses of RT affect the vasculature and immune cell infiltration in tumors of treated mice. Our results indicate that brachytherapy inhibits melanoma growth in a dose-dependent manner. Brachytherapy at a single dose of 10Gy had the greatest effect on changes in the levels of tumor-infiltrating immune cells. It most effectively reduced the level of protumorogenic M2 tumor-associated macrophages (TAMs) and increased the accumulation of cytotoxic CD8^+^ T lymphocytes. It changes an immunologically “cold” melanoma tumor (poorly infiltrated by immune cells) into a “hot” lesion with a robust immune infiltration. It seems that brachytherapy in a single dose of 10Gy is able to convert the tumor microenvironment of murine melanoma into an “in situ” vaccine. These data confirm the influence of RT doses on TME and constitute a promising avenue in anticancer therapy.

## 2. Results

### 2.1. Brachytherapy Inhibits the Growth of Murine Melanoma Tumors in a Dose Depending Manner

**Figure 1:**
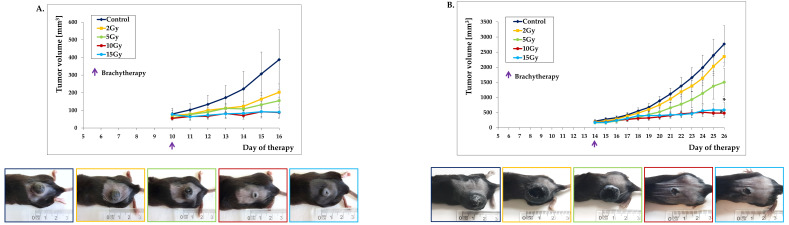

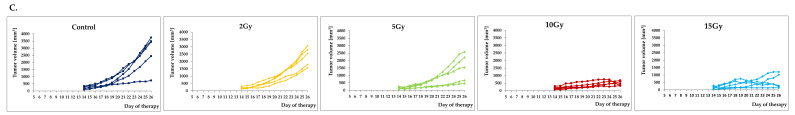
Inhibition of B16-F10 tumor growth in response to brachytherapy. Mice with well-developed tumors ((**A**): 60–70 mm^3^; *n* = 5; (**B**) 180–200 mm^3^; *n* = 5) were treated with different doses (2–15Gy) of brachytherapy. Brachytherapy in a single dose of 2Gy slightly inhibits the growth of B16-F10 tumors compared to the control group. A single dose of 5Gy reduced tumors growth compared to the 2Gy and untreated groups of mice. The most effective tumor growth inhibition was observed in the group of mice that received a brachytherapy in a single dose of 10Gy or 15Gy. * *p* < 0.003 compared to compared to 2Gy and control groups, the ANOVA followed by the Tukey’s post hoc test. Photographs were taken on the 16^th^ (**A**) and 28^th^ (**B**) days of therapy. (**C**) Individual tumor follow-up.

We studied the therapeutic effect of different doses of brachytherapy in murine melanoma tumors. The irradiation was performed in a shielded therapeutic room with a high-dose rate afterloader equipped with an iridium-192 radioactive source. The time of fraction delivery depended on the source activity (3–10 Ci). We used four single doses of irradiation: 2Gy, 5Gy, 10Gy, and 15Gy. Doses were planned using CT scans on the dedicated commercial platform. Irradiation was applied to well-developed tumors with a volumes of 60–70 mm^3^ (Figure 1A) as well as 180–200 mm^3^ (Figure 1B). In both cases, we observed the dose-dependent inhibition of murine melanoma tumor growth. A single dose of 2Gy only slightly inhibits the growth of B16-F10 murine melanoma tumors compared to the control group. We observed a more than 50% tumor growth inhibition following a 5Gy dose administration. The 10Gy and 15Gy doses inhibited tumor growth the most effectively (around 80%) compared to the other groups. However, increasing the dose from 10Gy to 15Gy did not improve the therapeutic effect (Figure 1). These data indicate that brachytherapy in a single dose of 10Gy is the most effective to inhibit murine melanoma tumor growth. Dose escalation did not improve the therapeutic antitumor potential of brachytherapy.

### 2.2. Brachytherapy Reduces Tumor Vasculature

**Figure 2:**
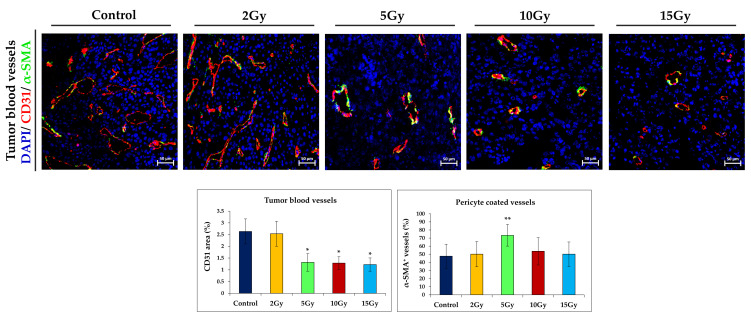
The effect of brachytherapy on tumor blood vessels in B16-F10 tumors. On the 16^th^ day of brachytherapy, mice were sacrificed and tumors were excised for the immunohistochemical staining of blood vessels. α-SMA and CD31 stainings were used to identify pericyte-covered tumor vessels (α-SMA^+^CD31^+^ vessels, percentage of CD31^+^ vessels; *n* = 4–5; 10 visual fields per tumor section; magnification 20×). Brachytherapy affects the structure and density of tumor blood vessels in treated mice in a dose-dependent manner (percentage of CD31^+^ vessels; *n* = 4–5; 10 visual fields per tumor section; magnification 20×). The doses of 5Gy to 15Gy reduce the area of vessels (CD31^+^) by about 50% compared to the control group. Significant changes in the vessel structure were also observed, but only after treatment with 5Gy the vessels had a thicker layer of pericytes (αSMA^+^) adjacent to their surface. * *p* < 0.00001 compared to 2Gy and control groups; ** *p* < 0.005 compared to other groups, Kruskal Wallis test.

Next, we attempted to investigate the effect of brachytherapy doses on the tumor vasculature. Tumor progression depends on its own vascular network, which, among other things, provides oxygen. The disruption of the tumor vasculature should lead to tumor growth inhibition [10,11]. We have observed that a dose of 2Gy does not induce any changes in tumor vasculature, both in density and vessel structure. The blood vessels, similarly to the control group, were mostly large and twisted, with an irregular shape and a thin wall. However, when a single dose of 5Gy was applied, we have observed a reduced tumor blood vessel density. Vessels showed a more regular shape, with a well visible layer of pericytes adhering to their surface (pericyte coverage was 20% higher compared to the control group). In the tumor sections, we observed fewer large blood vessels and more vessels with a wide lumen compared to the control group. Following the application of 10Gy or 15Gy, the vascular density was reduced by more than 50% compared to the control group. The vessels were mostly small, with a thick wall and a regular shape (Figure 2). These experiments indicate that there are no significant differences in tumor blood vessel density in groups receiving brachytherapy applied at single doses of 5Gy, 10Gy, and 15Gy. Only after applying a dose of 5Gy are more adherent pericytes observed.

### 2.3. Brachytherapy Changes the Phenotype of Tumor-Infiltrating Macrophages

**Figure 3:**
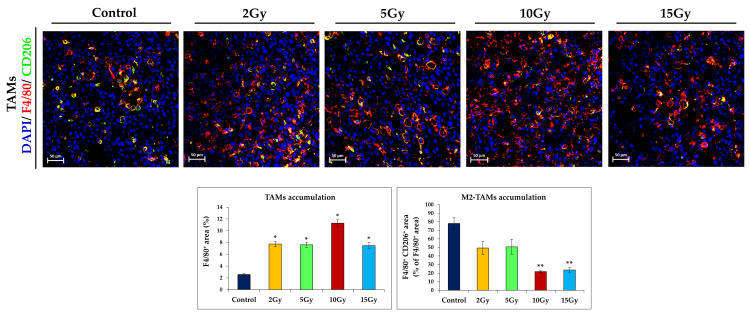
The effect of brachytherapy on the accumulation of tumor-associated macrophages (TAMs). On the 16^th^ day of therapy, the mice were sacrificed and tumors were excised for IHC analyzes. Tumors were stained with antibodies against CD206 and F4/80. Brachytherapy at a single dose of 10Gy has the greatest impact on the level of TAMs in B16-F10 tumors: the area of TAMs (F4/80^+^) increased more than four times, while the area of TAM M2 macrophages (F4/80^+^CD206^+^) was more than 3.5 times smaller compared to the control group. The areas of the TAM and M2 TAM macrophages were determined from three tumors per group; in each tumor 7 visual fields were analyzed (magnification 20×). * *p* < 0.003 compared to the control; ** *p* < 0.008 compared to the control, Kruskal Wallis test.

We have also examined the effect of brachytherapy on the presence and phenotype of tumor-associated macrophages (TAMs). Macrophages with M2 phenotype stimulate tumor growth. They participate in the formation of abnormal blood vessels and induce immunosuppression. In contrast, macrophages with the M1 phenotype stimulate naïve T cells to elicit a Th1/cytotoxic response. They participate in the normalization of tumor blood vessels and inhibit tumor growth [12,13,14]. We investigated the TAMs phenotype in the IHC (immunohistochemistry) sections obtained from tumors excised on the 16^th^ day of therapy. TAM infiltration (F4/80^+^) was increased three-fold in mice treated with 2Gy, 5Gy, or 15Gy brachytherapy doses compared to the control group. In contrast, a single dose of 10Gy increased the accumulation of TAMs in treated mice more than 4.5 times. Moreover, we observed a 1.5-fold decrease in the level of TAMs with the protumorogenic M2 phenotype (F4/80^+^/CD206^+^) in the tumors of mice treated with doses of 2Gy or 5Gy. However, following the application of 10Gy or 15Gy doses, the areas occupied by M2 macrophages decreased more than 3.5 times (Figure 3). In summary, a dose of 10Gy had the greatest impact on the changes in levels of tumor-infiltrating macrophages.

### 2.4. Brachytherapy Enhances the Infiltration of Lymphocytes into Tumor

**Figure 4:**
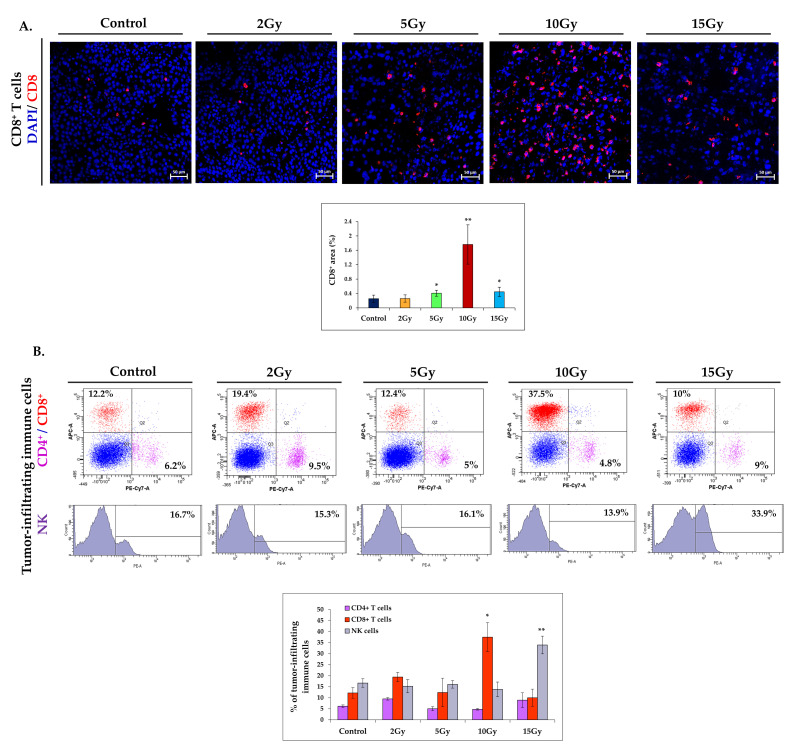
The effect of brachytherapy on the immune cells infiltration in the tumors. Six days after brachytherapy, the mice were sacrificed and tumor materials were collected for IHC and flow cytometry analyses. (**A**) Tumor cross-sections were stained with an antibody against CD8a. Brachytherapy at a single dose of 10Gy has the greatest effect on the infiltration of cytotoxic CD8^+^ lymphocytes in B16-F10 tumors. The level of tumor-infiltrating CD8^+^ T lymphocytes was increased almost sevenfold compared to the control group. The area of CD8^+^ lymphocytes was determined in four tumors per group; in each tumor, 10 visual fields were analyzed (magnification 20×). * *p* < 0.02 compared to 2Gy and control groups; ** *p* < 0.00001 compared to other groups, Kruskal Wallis test. (**B**) The level of T lymphocytes was determined in homogenous single-cell suspensions obtained from tumors (*n* = 4). The percentages of CD4^+^, CD8^+^ T lymphocytes, and NK cells were determined in the total viable CD45^+^ cells. Brachytherapy at a single dose of 10Gy increased the level of tumor-infiltrating CD8^+^ T lymphocytes (three times) compared to the control group. A higher level of NK cells was noted after a single dose of 15Gy brachytherapy. * *p* < 0.02 compared to the 5Gy, 15Gy, and control groups, Kruskal Wallis test; ** *p* < 0.007 compared to the other groups, the ANOVA followed by the Tukey’s post hoc test.

In the next stage of our work, we evaluated the effect of radiation doses on the infiltration of immune cells (CD4^+^, CD8^+^, and NK cells) in the tumors of treated mice. Tumor T lymphocytes infiltration is crucial in the anti-tumor immune response. T-cells secrete cytokines that affect the activation and differentiation of the immune cells, as well as they can directly destroy cancer cells. Cytotoxic CD8^+^ T lymphocytes have the ability to kill cancer cells. Their accumulation in tumors is a good prognostic factor. NK cells are cytotoxic innate lymphocytes which also play an important role in destroying cancer cells [15,16]. Using immunohistochemistry and flow cytometry, we investigated the phenotype of tumor-infiltrating lymphocytes following brachytherapy. We did not observe significant differences in the CD8^+^ T cells infiltration in the IHC tissue sections of 2Gy-irradiated tumors when compared to the control group. After doses of 5Gy or 15Gy, the accumulation of CD8^+^ T lymphocytes was twice increased. However, the largest recruitment of CD8^+^ T cells was visible after the 10Gy dose. Area occupied by cytotoxic CD8^+^ lymphocytes increased almost 7-fold compared to the control group (Figure 4A). The cytofluorometric analyses of single-cell suspensions obtained from tumors collected after brachytherapy showed slight differences in the tumor-infiltrating CD4^+^, CD8^+^ and NK cells levels compared to the control tumors. The exceptions were tumors irradiated with the 10Gy dose, where the infiltration of CD8^+^ T lymphocytes increased three times compared to the control group. In addition, the tumor-infiltrating NK cells level was doubled after the 15Gy dose when compared to the control group (Figure 4B). These experiments indicate that brachytherapy at a single dose of 10Gy the most effectively increases the level of tumor-infiltrating cytotoxic CD8^+^ T lymphocytes.

## 3. Discussion

Radiotherapy is used for therapeutic or palliative purposes in more than 50% of cancer patients [7]. Irradiation induces a number of different types of cell death that depend on the cell genetic background, radiation dose, and the surrounding tumor microenvironment [8]. TME plays a key role in the effectiveness of radiation therapy. A tumor is not only a mass of transformed cells, but a “new organ” composed of various non-malignant cells that may constitute a large proportion of the tumor mass. Among these cells are, namely, vascular endothelial cells, fibroblasts, adipocytes, pericytes, and the main players—immune cells [17]. There are no conclusive data indicating what dose of radiation induces or suppresses the anti-cancer immune response. The literature data show contradictory information—both LDR and HDR can activate and also inhibit the anti-tumor immune response [9].

Therefore, as part of our research, we focused on the effect of various doses of radiotherapy—2Gy, 5Gy, 10Gy, and 15Gy—on the inhibition of B16-F10 murine melanoma tumor growth. In this brief study, we examined how selected doses of RT affect the vasculature and immune cell infiltration in the tumors of treated mice compared to control mice. In our work, we used contact radiotherapy (brachytherapy), which is used in the treatment of skin cancer [5]. Brachytherapy is a treatment method as old as radiation therapy [18] and has been described as the first form of conformal radiation therapy. Brachytherapy is a radiation method in which radioactive sources are placed within or very close to the tumor. It allows a high cancer to normal tissue dose ratio [19]. The success of brachytherapy has increased with technological progress, which allows CT and MRI imaging and the individual planning of radiation dose distribution for a given patient. Despite the use of brachytherapy for decades, there are very little data on the biological processes that occur in a tumor subjected to this therapy. Furthermore, there are no data on how brachytherapy doses affect the tumor microenvironment.

The International Commission on Radiation Units and Measurements defined three categories of brachytherapy dose rate: low (LDR)—range: 0.4 to 2 Gy/hr; medium (MDR)—range: 2 to 12 Gy/hr; and high (HDR)—more than 12 Gy/hr [19]. We conducted studies with the use of LDL, MDL, and HDL doses and tested how the doses affect TME. According to the literature, the dose effect may be disparate. Klug et al. [20] have shown that a low dose of gamma irradiation (LDI; with a Gammatron Cobalt 60 therapy unit) normalizes the tumor blood vessels and reprograms macrophages towards the anti-tumor iNOS+/M1 phenotype, which recruits cytotoxic T lymphocytes to the tumor. However, our results indicate that a single dose of 2Gy does not influence either tumor blood vessel density or immune cells infiltration. We also have not observed a significant inhibition of tumor growth after low dose radiation. LDR mainly induces the apoptosis of cancer cells. Antigen-presenting cells are not activated, but TAM M2 and MDSC immunosuppressive macrophages are recruited [9]. The used dose of 2Gy does not trigger any changes in tumor vasculature, either in density or structure. The majority of vessels, similarly to the control group, were large and twisted, with an irregular shape and thin walls. Endothelial cells survive the conventional dose of 2Gy, which stimulates angiogenesis and neovascularization processes [21]. Heissig et al. [22] have shown that low-dose radiation (Caesium Gy source) promotes tissue revascularization via mast cells-mediated VEGF (vascular endothelial growth factor) release and progenitor cell mobilization through MMP-9.

In this study, the 5Gy and 10Gy doses were tested as MDR. A single dose of 5Gy reduced the tumor mass of mouse melanoma by approximately 50%. In those irradiated tumors, we observed a reduced blood vessel density. The vessels exhibited a more regular shape and a 20% higher pericyte coverage compared to the controls. In tumor sections, we observed blood vessels with wider lumen compared to the control tumors. The increased presence of pericytes in the 5Gy dose-irradiated tumors may be due to the vessels changing into structures similar to normal ones. Tumor vasculature normalization reveals as a blood vessel density reduction, more regular vascular distribution, increased pericyte coverage, and tumor perfusion [13,23,24]. However, some papers report that the increased presence of pericytes may be a poor prognostic factor. Elevated pericyte coverage has been associated with aggressive melanoma and renal cell carcinoma, treatment resistance, and adverse patient outcomes [25]. Moreover, in vitro models indicate that ≤ 5Gy doses may stimulate angiogenesis and/or vasculogenesis in endothelial cells [26]. In addition, after a 5Gy dose, we observed a three-fold increase in TAMs infiltration and a slight decrease in TAMs with the M2 phenotype compared to the control group. There was no significant difference in the infiltration of CD4^+^, CD8^+^, and NK cells as compared to the control group. These results indicate that the therapeutic effect of the 5Gy dose is due to a reduction in the number of tumor blood vessels that are necessary for cancer progression. Destroying tumor blood vessels and blood flow reduction lead to a decrease in tissue oxygenation, which indirectly induces cell death and, as a result, reduces tumor volume [9].

After applying a single dose of 10Gy, the tumor vasculature was also reduced by a half. Most of the vessels were small, with thick walls and regular shape. However, unlike the other tested doses of brachytherapy, a single dose of 10Gy had the greatest effect on changes in the levels of tumor-infiltrating immune cells; the accumulation of TAMs increased more than 4.5 times and the area occupied by M2 macrophages decreased more than 3.5 times in the tumors of treated mice. Additionally, we observed the largest CD8^+^ T lymphocytes infiltration in tumors treated with the 10Gy dose compared to the other groups. The area occupied by the CD8^+^ T cells increased almost seven times when compared to the control group. The 10Gy dose inhibited tumor growth the most effectively (around 80%) compared to other groups. We assume that the therapeutic efficacy of a single dose of 10Gy of brachytherapy is due to the reduced tumor vascular density but also due to the reduced accumulation of pro-tumor M2 macrophages and the robust recruitment of CD8^+^ T lymphocytes in the tumors of treated mice. An activated by a 10Gy antitumor immune response is most likely caused by the “in situ” vaccination effect. Formenti and Demaria [27] indicated that in some cases radiotherapy may successfully immunize the patient against cancer, converting the irradiated tumor microenvironment into an “in situ” vaccine and providing the host with powerful tools to fight against cancer. RT induces immunogenic cancer cell death, which results in calreticulin (CRT) exposure on the cells surface and the release of tumor neoantigens as well as death-associated molecular patterns (DAMPs; among others, adenosine triphosphate (ATP) and high mobility group box 1 (HMGB1) protein). These signals enable the recognition of dying cells by dendritic cells (DCs), which subsequently phagocytize them. They also induce the maturation of DCs, enabling a more effective antigen presentation to T lymphocytes. The activated DCs migrate to lymph nodes and activate naïve T lymphocytes. Lymphocytes, stimulated by antigens specific to tumor cells, become cytotoxic effector cells. These effector T cells, attracted by the RT-induced chemokines release, migrate to the tumor, where they recognize and destroy cancer cells [28,29]. Anticancer therapy that transforms the tumor from “low intratumoral T cell infiltration type” to a “T cell inflamed phenotype” increases the likelihood of patients responding to checkpoint blockade therapy. Therefore, “in situ” vaccination represents one of the most promising strategies [30].

In our research, we also have tested HDR—15Gy. Similar to the 10Gy dose, we observed an 80% inhibition of mouse melanoma tumor growth. However, after the application of 15Gy, a radiation-induced skin reaction appeared on the back of treated mice (data not shown). Like the application of the 5Gy or 10Gy doses, the area of tumor vessels was reduced by more than 50% in the tumors of treated mice. There were also no differences in the TAMs infiltration in tumors exposed to 15Gy brachytherapy compared to the 2Gy or 5Gy-treated mice. As with 10Gy, the area occupied by M2 macrophages was reduced more than 3.5 times. The cytofluorometric analyses of tumors excised after brachytherapy showed slight differences in the infiltration of CD4^+^ and CD8^+^ T lymphocytes compared to the control group. In contrast, the NK cell infiltration was increased twice in tumors irradiated with the 15Gy dose when compared to the control group. Irradiation should increase the cytotoxicity of NK cells against cancer cells [31]. However, Finkel et al. [32] have demonstrated the dual role of NK cells in the anti-tumor response caused by radiation. On the one hand, upon the activation of the anti-tumor response, NK cells are important for killing cancer cells. On the other hand, NK cells may be unfavorable during immune activation because they can have a negative effect on the formation of T-cell-mediated immunity. NK cells in the tumor microenvironment may induce lysis by the degranulation of T lymphocytes. Hence, we could observe slight differences in the CD8^+^ lymphocyte tumor infiltration compared to the other groups and could not observe a further enhanced anti-tumor therapeutic effect. To sum up, increasing the dose from 10Gy to 15Gy did not improve the therapeutic antitumor potential of brachytherapy.

## 4. Materials and Methods

### 4.1. Cell Line, Mice, and Ethics Statement

The B16-F10-Luc cell line (murine melanoma, ATCC, Manassas, VA, USA) was maintained using RPMI (Gibco BRL, Paisley, UK) supplemented with 10% heat-inactivated FBS (Gibco BRL). The cell cultures were maintained under standard conditions (37 °C, 5% CO_2_, 95% humidity) and passaged twice a week.

Mice (six-to-eight-week old) C57Bl/6NCrl females (Charles River Laboratories, Wilmington, MA, USA) were housed in Maria Sklodowska-Curie National Research Institute of Oncology, Gliwice Branch (Poland). The mice were kept under a 12 h dark/12 h light cycle in an SPF (Specific Pathogen-Free) animal facility. The experimental protocol was approved by the Local Ethics Commission (Medical University of Silesia, Katowice, Poland (permit number: 21/2020; 6 April 2020). The study was carried out in strict accordance with the recommendations in the Guide for the Care and Use of Laboratory Animals of the National Institutes of Health. All efforts were made to minimize animal suffering by qualified personnel [13]. All the experiments on animals were conducted in accordance with the 3R rule. The mice were inoculated with B16-F10-Luc tumor cells and were treated as described above. Each procedure was terminated by cervical dislocation and tumor collection for the immunofluorescence analysis. Microscopy and cytometric analysis were performed as described below.

### 4.2. Inoculation of Animals

C57Bl/6NCrl mice were injected subcutaneously with 2 × 10^5^ B16- F10-Luc cells in 100 μL of PBS¯. The mice with developed tumors were randomly divided into experimental groups, both to the treated and control groups. In all the experiments, the number of mice in each group was 5. The growing tumors were measured with calipers and the tumor volumes were determined using the formula: volume = width^2^ × length × 0.52. The mice were weighed during the monitoring of the sizes of the tumors. After 10 or 14 days, brachytherapy was performed. Monitoring for animal health was performed every day (activity, appetite, behavior, and response to treatment) [13].

### 4.3. Contact Radiotherapy (Brachytherapy)

Mice with well-developed tumors (60–70 mm^3^ or 180-200 mm^3^; *n* = 5 per group) were treated with contact radiotherapy (brachytherapy). Brachytherapy was used at doses of 2Gy, 5Gy, 10Gy, or 15Gy (acc. to International System of Units the gray (Gy) is the unit of absorbed radiation dose of ionizing radiation (equal to one joule per kilogram)). Brachytherapy was performed with a dedicated applicator, which was placed directly in the tumor area. The dose per fraction was planned to be specified 2–3 mm from the applicator surface. The dose was adjusted to the tumor thickness to avoid unwanted dose coverage in the organs at risk beyond the tumor. The dose was planned using CT scans on the dedicated commercial platform. Irradiation was performed in the shielded therapeutic room with a high-dose rate afterloader equipped with an iridium-192 radioactive source (Microselectron, Nucletron) in the Brachytherapy Unit, Maria Sklodowska-Curie National Research Institute of Oncology, Gliwice Branch (Poland). The time of fraction delivery was decided depending on the source activity (3–10 Ci). It was recalculated with dedicated software every time. The surface applicator was placed and fixed in the tumor area (Pehahaft) before every fraction. The transfer tubes were connected and irradiation was performed. The applicator was detached immediately after irradiation.

### 4.4. Isolation and Identification of Tumor-Infiltrating Lymphocytes and NK Cells

The tumors were collected and minced manually into small pieces. A single-cell suspension from tumors were obtained by passing them through the 70 µm and 40 µm cell strainers (BD Biosciences, San Jose, CA, USA). Red blood cells were lysed using a 0.15 M ammonium chloride solution. Dead cells were removed by centrifugation using the Lympholyte-M gradient (Cedarlane, Ontario, Canada). 7-AAD (7-aminoactinomycin D) Viability Staining Solution (BioLegend, San Diego, CA, USA) was used to stain nonviable cells 10 min before running the flow analysis. To identify the subpopulations of T lymphocytes and NK cells, the following antibodies were used: FITC-CD45, PE-Cy7-CD4, APC-CD8, and PE-CD49b (BioLegend). In the flow cytometric analyses (BD FACSCanto, BD, Franklin Lakes, NJ, USA), the gates dividing negative from positive cells were based on isotype antibody control probes [33].

### 4.5. Immunohistochemical Analysis of Immune Cells and Tumor Vasculature

The tumors were collected, frozen in liquid nitrogen, and sectioned into 5 μm slices. Macrophages in the tumor sections were stained with anti-F4/80 and anti-CD206 antibodies (Abcam, Cambridge, UK), followed by appropriate fluorochrome-conjugated (Alexa Fluor 594 (Abcam) and FITC (Vector Laboratories, Burlingame, CA, USA) secondary antibodies. The cytotoxic T lymphocytes were stained with anti-CD8α (Abcam) and subsequently with Alexa Fluor 594-conjugated secondary antibodies (Abcam). For the immunohistochemical analyses of pericytes coverage, tumor blood vessels sections were incubated with anti-α-Smooth Muscle Actin and anti-CD31 antibodies (Abcam) and subsequently with Texas Red and FITC-conjugated secondary antibodies (Vector Laboratories) [13]. All the sections were mounted in VECTASHIELD Mounting Medium with DAPI (Vector Laboratories). Microscopic observations were performed using a LSM710 confocal microscope (Carl Zeiss Microscopy GmbH). The obtained confocal images were analyzed with ImageJ 1.48v (National Institutes of Health and the Laboratory for Optical and Computational Instrumentation, LOCI, University of Wisconsin, USA) and the results were expressed as the percentage of area [%].

### 4.6. Statistical Analysis

The statistical significance of differences between the experimental and control groups in the tumor growth kinetics and immunofluorescences analyses was evaluated using difference tests. The distribution normality was tested using the Shapiro–Wilk test. The homogeneity of variance was checked by the Brown–Forsythe test. To compare several study groups, an ANOVA (with the Tukey post-hoc test) or the Kruskal–Wallis (with a multiple comparison of average ranks for all trials test) test was used. The statistical analysis was performed using the Statistica 12 software. *p*-values *< 0.05* were considered statistically significant.

## 5. Conclusions

Our brief report indicates that a single dose of 10Gy reduces the tumor blood vessel density and leads to the development of a robust immune infiltration. Perhaps a single dose of 10Gy “unmasks” the tumor, making it visible to the immune system. It converts an immunologically “cold” tumor (murine melanoma that is poorly infiltrated by immune cells) into a “hot”’ lesion. An activated antitumor immune response under this dose is most likely caused by the “in situ” vaccination effect. This leads to a significant inhibition of tumor growth. Our data indicate that brachytherapy in a single dose of 10Gy is a promising avenue in anticancer therapy.
